# In This Issue

**DOI:** 10.1002/cfg.78

**Published:** 2001-04


**Comparative and Functional Genomics**
*Comp Funct Genom* 2001; **2**: 57–58.
**DOI**: 10.1002/cfg.78

### In this issue

### Organ laterality mutants in zebrafish

Jau-Nian Chen and colleagues have previously identified a panel of zebrafish mutants showing abnormal left-right positioning of one of more organs, combined with perturbed gastrulation, body form or midline structures. In this study they have identified seven new mutants, which, unlike the earlier ones, are apparently normal at the embryonic stages examined (apart from the organ placement effects). Using probes to mark the locations of the early primordia of the heart, gut, liver and pancreas, they have further characterised the seven new mutants and a representative subset of the earlier mutants.

### DNA-array comparison of a wine yeast and a laboratory strain

Hauser *et al.* have used a yeast DNA-array in a first scan of the genetic differences between a wine yeast strain (T73) and a laboratory strain derived from S288c. Under standard growth conditions they identified over 40 genes that showed significantly different expression between the two strains. Some of these differences in expression were verified by Northern blot analysis and sequencing of the promoter regions of two of these genes identified differences that might explain the variation in expression level between the two strains.

### The complete mitochondrial genome of *Yarrowia lipolytica*

In their paper, Kerscher *et al.* describe the complete genome sequence of the mitochondrion of the obligate aerobic yeast *Yarrowia lipolytica*. All of the genes are on the same strand and use the typical mold mitochondrial genetic code. Its gene content is typical of animal and higher fungal mitochondria, encoding hydrophobic subunits of respiratory chain complexes, large and small ribosomal RNAs, and tRNAs. However, there is no gene for a tRNA capable of reading CGN (arginine) codons. There is a coincident lack of CGN codons in the exonic open reading frames, although they do appear in the intronic open reading frames, some of which appear to be pseudogenes.

### Featured Organism: *Arabidopsis thaliana*

The completion of the sequence of the first plant genome (*Arabidopsis thaliana*) was announced in December 2000. We feature this important model dicot, which has much to offer, not only to those working on dicot crops, but also to researchers studying monocots. With its small genome, and indeed small size, *Arabidopsis* is highly amenable to functional analyses, and, coupled with the knowledge of every gene encoded by this plant, we expect that this will soon result in an explosion of functional data on plant genes. Prof. Pamela Green and Dr Sean May share their enthusiasm for this new phase in plant genomics with us.

### Interview with Michael Bevan: The *Arabidopsis* genome is sequenced, what next?

Professor Michael Bevan was the co-ordinator of the European Union *Arabidopsis* Genome Sequencing Consortium. He is a member of GARNet and UK Crop.Net, two important UK networks involved in *Arabidopsis* functional genomics and providing plant bioinformatics resources, respectively. He has also been appointed the co-ordinator of a new EU funded consortium called EXOTIC, which aims to study the expression patterns of some 5000 *Arabidopsis* genes. He talked to us about the approaches these research teams will be using to increase our knowledge of plant gene function, and some of the directions he thinks the field will take, now that the *Arabidopsis* genome sequence has been completed.

### Website review: Structural genomics on the web

The application of X-ray crystallography and nuclear magnetic resonance spectroscopy to the

Copyright © 2001 John Wiley & Sons, Ltd.

determination of protein structures is certainly not a new idea, however, this field is changing and gathering pace before our eyes. Structural data can add much to the knowledge on a chosen protein or protein family, including the potential for inference of biochemical function. New consortia are forming to work on ways of increasing the throughput of these approaches, and to study the protein structure sets of selected organisms. We present a guide to websites providing software tools and databases for structural genomics, and the homepages of selected consortia in the field. We also provide examples, for less experienced users, of what can be gleaned from these valuable resources.

The Genomics Website at Wiley www.wiley.co.uk/genomics

The **Genomics** website at Wiley is a **DYNAMIC** resource for the genomics community, offering **FREE** special feature articles and new information **EACH MONTH**.

Find out more about *Comparative and Functional Genomics*, and *Proteomics*, and how to view many articles **FREE OF CHARGE**!

Visit the **Library** for hot books in Genomics, Bioinformatics, Molecular Genetics and more.

Click on **Journals** for information on all our up-to-the minute journals, including: *Genesis*, *Bioessays, Gene Function and Disease, Human Mutation, Genes, Chromosomes and Cancer* and the *Journal of Gene Medicine.*

Let the **Genomics** website at Wiley be your **guide** to genomics-related web sites, manufacturers and suppliers, and a calendar of conferences.

WILEY

2171

Copyright © 2001 John Wiley & Sons, Ltd. 

